# Supplementary material for: Challenges to generating political prioritization for adolescent sexual and reproductive health in Kenya: A qualitative study
Source: PLoS One. 2019 Dec 19;14(12):e0226426. doi: 10.1371/journal.pone.0226426 (PMC6922405; doi:10.1371/journal.pone.0226426)
Supplement: S1 File — (DOC) [file pone.0226426.s001.doc]

**S1: Consolidated criteria for reporting qualitative research (COREQ) checklist**

Tong A. Sainsbury P. Craig J. Consolidated criteria for reporting qualitative research (COREQ): a 32-item checklist for interviews and focus groups. International Journal for Quality in Health Care. 19(6): 349-357

| **Item** | **Response** | **Location in manuscript (section, page number)** |
| --- | --- | --- |
| ***Domain 1: Research team and reflexivity*** | | |
| *Personal Characteristics* | | |
| 1. Interviewer/ facilitator | Experienced interviewers/moderators | Methods, page 5 |
| 2. Credentials | MAO holds MBChB, MSc and is PHD candidate; GR holds an MD and MPH, JSW holds a PHD, DO is MBChB, EG has an MD EB is MBChB, MMeD, MPH, PhD, MBE, CIP; CB has a PhD, | Title Page (Information on credentials is available only for MAO) |
| 3. Occupation | MO is a medical doctor and Global Health Research Scientist, GR is a professor in epidemiology, Program Coordinator, EB is Chief Research Officer & Co-Director of Research Care Training Program; JSW occupation; DO is a program facilitator for sexual reproductive health UNFPA Kenya; CB is a professor of paediatrics and obstetrics at UCSF, EG is the director of the UCSF Centre for Implementations sciences | (Information on author occupation is not available in the manuscript, only affiliations available) |
| 4. Gender | MO, EB and CB are females, GR, JW, DO and EG are males |  |
| 5. Experience and training | All authors are technically and methodologically experienced researchers in Kenyan settings | Methods page 5, Title Page |
| *Relationship with participants* | | |
| 6. Relationship established | Some participants might have had previous working relationship with MO and EB through interactions in technical working groups | Methods, pages 4-5 |
| 7. Participant knowledge of the interviewer | Participants were informed about the reasons for the research via the invitation to participate and the consent form. | Methods, pages 4-5 |
| 8. Interviewer characteristics | No interviewer-related biases identified. |  |
| ***Domain 2: Study design*** | | |
| *Theoretical framework* | | |
| 9. Methodological orientation and theory | Thematic analysis approach. The coding framework based on literature, topics from interview guides, and emerging themes from transcripts. | Methods, page 5 |
| *Participant Selection* | | |
| 10.Sampling | Participants were purposively selected for maximum variation in representation across different sectors. | In-depth interviews: Methods, page 5 |
| 11. Method of approach | The lead researcher and a representative from the Ministry of health, division of reproductive and maternal health identified potential participants. Participants were then contacted via phone call and given a brief overview of the study and asked if they were willing to participate. | In-depth interviews: Methods, page 4-5 |
| 12. Sample size | 14 National state and non-state actors from government, civil society, donor organisations, religious society, youth and education sector, health officials | Methods, page 4 |
| 13. Non-participation | All eligible and approached individuals agreed to participate in the study. | N/A |
| *Setting* | | |
| 14. Setting of data collection | Interviews were conducted in private setting at the participants choice of place | Methods, page 5 |
| 15. Presence of non-participants | No | N/A |
| 16.Description of sample | National state and non-state actors, > 18 years of age involved in adolescent health policy making  All participants provided their consent to participate in the study. | Methods, page 4 |
| *Data collection* | | |
| 17. Interview guide | Qualitative in-depth interview guides were developed based on a review of the literature and our team’s prior studies on adolescent sexual reproductive health. Ultimately we adapted a guide that had been used before in similar settings. The guide related and included questions on 1) the current priority for adolescent SRH in the health agenda of Kenya, 2) how adolescent SRH fit in with the key health priorities for Kenya, 3) who is responsible for setting major national health policy and who holds significant influence over these decisions, 4) what sources within Kenya, if any, provided pressure on policy makers to increase resource allocation for adolescent SRH, and 5) how adolescent SRH should be framed to political leaders in order to generate political support. The interview guide was reviewed and approved by appropriate ethics review boards. | Methods, page 5; Ethics approval and consent to participate, page 5 |
| 18. Repeat interviews | N/A | N/A |
| 19. Audio/ visual recording | All interviews were audio recorded. Audio-recordings were transcribed, translated and coded using Dedoose software. Interviews were digitally recorded and transcribed verbatim by professional transcriptionists, excluding any identifying information. All files were password-protected and stored in a secure location. | Methods, page 5 |
| 20. Field notes | Interviewers took notes for purposes of assistance with transcription. | Methods. Page5 |
| 21. Duration | Each interview lasted approximately 1 -1.5 hours. | Methods, page 5 |
| 22. Data saturation | Data saturation was discussed and it was concluded that the data saturation was reached. | Methods, page 4 |
| 23. Transcripts returned | N/A | N/A |
| ***Domain 3: Analysis and findings*** | | |
| *Data analysis* | | |
| 24. Number of data coders | A team of two researchers coded the transcripts. Consistency of coding between two ndividuals was established by initially coding the same transcripts and through frequent discussion between coders until consistency was fully established. Excerpts from broad codes were then fine-coded using an inductive approach by the same coders. All authors reviewed the qualitative report and final themes. | Methods, page 5 |
| 25. Description of coding tree | Thematic analysis approach was utilized. The coding framework based on Shiffman and Smith literature, topics from interview guides, and emerging themes from transcripts. | Methods, page 5 |
| 26. Deviation of themes | The coding framework based on the Shiffman and Smith conceptual framework as well as literature, topics from interview guides, and emerging themes from transcripts. | Methods, page 5; Table 1, page 20 |
| 27. Software | Dedoose qualitative software program (Sociocultural Research Consultants, LLC) was utilized. | Methods, page 5 |
| 28. Participant checking | Findings are not yet disseminated |  |
| *Reporting* |  |  |
| 29. Quotations presented | Participant quotations are provided to illustrate the themes and attributed to participants. De-identified participant characteristics are provided for each quote to distinguished between type gender and state and non state actors | Results, pages – 6-12 |
| 30. Data and findings consistent | Yes | Discussion, pages – 12-15 |
| 31. Clarity of major themes | Four major themes are clearly presented with rich narratives | Results, pages– 6-12; Table 1, page 20 |
| 32. Clarity of minor themes | Minor themes were in line with the Shiffman and Smith conceptual framework are discussed | Results, pages– 6-12; Table 1, page 20 |
