# Supplementary material for: Challenges to generating political prioritization for adolescent sexual and reproductive health in Kenya: A qualitative study
Source: PLoS One. 2019 Dec 19;14(12):e0226426. doi: 10.1371/journal.pone.0226426 (PMC6922405; doi:10.1371/journal.pone.0226426)
Supplement: S2 File — (DOC) [file pone.0226426.s002.doc]

**S1:** **Semi-structured interview guide**

**Introduction.**

Hello, my name is _________________. I am a researcher working on a study on the priority setting process for ASRH**.** I am asking you to participate in this research study because you have the necessary information that will be key in completing my project. The overall purpose of this study is to help broaden the understanding of the priority setting processes for ASRH after the Maputo protocol ratification using Kenya as a case study. The questions that you will be asked will touch on the following areas:

1. Personal information including occupation and roles in implementation of government policies.
2. Health care priorities in Kenya, sexual reproductive health, HIV prevention, National Health policies, Millennium developmental goals and Health related data and their use.

You will be interviewed for approximately 60 minutes and you will be digitally recorded and transcribed. You may skip any question that makes you uncomfortable. You may also stop the interview/survey at any time.

**General**

1. What are the main health care priorities in Kenya currently at a national level?
2. Why have these areas received attention do you think?
3. What are the key priorities in Kenya in health at present in your own opinion?
4. How does ASRH fit or not fit in with the key health priorities for Kenya?
   1. Why is this?
5. Do you think that the ministry of health and the ministry of finance correctly aligns their policies and funding with the priority health areas in Kenya?
6. If you could double your health budget (or for local informants, your hospital budget) from next year, where would you spend the additional money?
7. What is the current priority for scale-up of ASRH capacity in the health agenda of Kenya?
   1. How important is providing ASRH compared to other health initiatives such as preventing HIV or decreasing maternal deaths?
8. Please tell us about Kenya’s (or county’s) major successes and challenges with providing ASRH.
9. How do you see ASRH contributing to these priorities and helping to achieve your country's health goals?
10. What is required to see ASRH as a greater priority to your healthcare system?

**Actor Power**

1. Who is responsible for setting major national health policy and who holds significant influence over these decisions? Both within Kenya and internationally?
   1. Is there agreement over the general direction in which to invest in the health sector?
2. To what extent have the health priorities in Kenya been influenced by;
   1. Locally articulated health needs? (i.e. through local advocacy, local and national politics etc.)
   2. Donor driven agendas (public, private, non-governmental organizations (NGOs), bilateral, multilateral etc.)
   3. The Maputo protocol and the African continental framework on sexual reproductive health
   4. The Millennium Development Goals (MDGs) and Sustainable Development Goals (SDGs) agenda
   5. Others?
3. What sources within Kenya, if any, have provided pressure on policy makers to increase ASRH capacity? Internationally?
   1. How effective have they been in achieving their goals?
   2. How do you regard and utilize the World Health Organisation (WHO) recommendations for ASRH services?
   3. How do these recommendations support or differ from how you would like to see ASRH implemented within Kenya?
   4. How are local and national leaders similar or different in their roles for promoting ASRH?
4. Are there any points of cohesion between internal actors and external actors in ASRH?

**Ideas**

1. Internal Frame
   1. What key ASRH interventions are most important for Kenya?
   2. What are the major ASRH issues in Kenya?
      1. *If subject does not see certain issues as ‘ASRH’* – Why do you not consider [issue] as an ASRH issue / disease? (Maternal/perinatal issues for example)
   3. How does the community view ASRH's strengthening role in overall health system strengthening?
   4. Can ASRH be provided in Kenya at a low cost?
2. External Frame
   1. How should the issue of providing ASRH be framed to political leaders in order to generate political support?

**Political Contexts**

1. Is providing ASRH on the national or local or hospital health agenda in Kenya?  How did it get there?
2. How is health care funded within Kenya?
   1. Public/private/NGO sectors?
   2. Out of pocket payment
3. What role does each sector play in providing healthcare (or ASRH) in Kenya?
4. How much of the health budget, if any, is explicitly allocated toward providing ASRH in Kenya?
5. Have there been any opportunities you are aware of within the national or local political/health agenda when ASRH may have gained more traction? e.g. during a focus on maternal health or HIV?
   1. Was this opportunity utilised? Why/why not?
   2. Who drove this or should have driven this in your opinion?
6. Are there mechanisms for advocating for health issues/priorities at a local and national level political level?
   1. What are these?
   2. Are they effective in bringing about change (responsiveness)? Why? /Why not?
   3. How could they be more effective?

**Issue Characteristics**

1. To what extent are you able to use local and national health data to inform planning and resource allocation in health?
2. What tensions exist between pursuing short and long-term strategies in building ASRH capacity?
3. What are the major points of intervention for increasing ASRH capacity in Kenya
4. How are health priorities addressed in Kenya? Vertical vs. diagonal vs. horizontal strategies?
5. What, if any, are the main health indicators you focus on?
   1. Are any of these ASRH?
6. Are you aware of the burden of Adolescent health in Kenya?
   1. Trauma/injuries?
   2. NCDs
   3. Maternal health
   4. Where do these diseases stand in relation to ASRH?
7. What would you consider the top 10 diseases in Kenya (rank them)?
8. What are the most significant ASRH issues in Kenya?
   1. How important is providing subspecialty ASRH in Kenya?
9. What do you think are the main reasons why improving ASRH capacity is not given high priority?
10. What are the barriers to deliver ASRH care in Kenya?
11. How do the requirements needed for delivering ASRH services in Kenya differ from those of infectious diseases, like HIV, Malaria?
    1. What are the infrastructure requirements and gaps?
12. Who should be providing ASRH?
    1. What are the barriers or bottlenecks to adequate human resources for ASRH in Kenya?
13. What kinds of focusing events shape policy attention for health causes?
14. What are the features of focusing events that give them agenda setting power?
15. Under what conditions do indicators have agenda setting power?
16. Under what conditions do they fail to have impact?
17. Under what circumstances can/do domestic political entrepreneurs make a difference?
18. What is it they do that makes a difference?
19. What features of international health policy networks give them the capacity to influence domestic health priorities?
20. In particular, what is the relationship between network structure and the power to influence?
21. As donor–government relations in health are so frequently contentious, under what circumstances is productive cooperation likely to emerge?
