# Supplementary material for: Challenges to generating political prioritization for adolescent sexual and reproductive health in Kenya: A qualitative study
Source: PLoS One. 2019 Dec 19;14(12):e0226426. doi: 10.1371/journal.pone.0226426 (PMC6922405; doi:10.1371/journal.pone.0226426)
Supplement: Attachment — Submitted filename: Response to Reviewers_25NOV2019.doc [file pone.0226426.s004.doc]

25 November 2019

Thank you for your time and careful review of our manuscript “PONE-D-19-27067 Challenges to generating political prioritization for adolescent sexual and reproductive health in Kenya: a qualitative study.” We deeply appreciate the feedback from the reviewers.

We have addressed your comments in the revised manuscript and have detailed our responses to each point below (in blue font).

Thank you very much for your time and consideration of this revised manuscript for publication in *PLOS ONE*. If there is any additional information or details about the study that we can provide, please do not hesitate to contact me. We look forward to your response.

Sincerely,

Authors

**Review Comments to the Author**

Please use the space provided to explain your answers to the questions above. You may also include additional comments for the author, including concerns about dual publication, research ethics, or publication ethics. (Please upload your review as an attachment if it exceeds 20,000 characters)

**Reviewer #1: The authors have addressed the issues raised by reviewers. There are, however, a few editorial corrections to make. These include:**

1) Study setting, second statement (page 6): Rephrase to read: “At the global level, these include …” Also, delete “of 2002” at the end of the UN General Assembly on Special Session on Children, and “at a global level” at the end of the statement since it is unnecessary repetition of the phrase used at the beginning. This has been edited

It now reads, “At the global level these include the 1994 United Nations at the International Conference on Population and Development (ICPD) [26], the 2002 United Nations General Assembly Special Session on Children [27]…”

2) Study setting, third statement (page 6): Rephrase to read: “At the global level …” and delete one “the” after “adopted” to avoid unnecessary repetition. This has been edited
It now reads, “At the regional level, in Africa, Kenya has adopted the Maputo Protocol”
3) Study setting, fourth statement (page 6): Insert “the” before “National Youth Policy” for the statement to read well. This has been edited
It now reads, “Locally the 2010 Constitution of Kenya, the National Youth Policy (2007)…”

4) Ideas: Framing the problem, second quote (pages 14-15): Based on the authors’ response to earlier comment regarding the clarity of the quote, they need to add “[comprehensive sexuality education]” in brackets as shown after “invest more” so that it is clear that the participant was referring to investing in comprehensive sexuality education rather than in programs to prevent pregnancy among adolescents generally. This has been edited

It now reads, “…*So I think that both culturally and religiously, there is that feeling that if you invest more [in comprehensive sexuality education] then, they will now know that it is their right (State Corporation; IDI_I0).”*

5) Issue characteristics, fourth quote (pages 19-20): Based on the authors’ response to an earlier comment regarding the quote, it should then read: “And the reason for failure ...” This has been edited
It now reads, “*I think that is where the challenge lies. And the reason for failure to optimize the services for young people is actually because resources are not there….”*

6) Political contexts, first quote (pages 20-21): Based on the authors’ response to the comment on this quote, if the participant used the word “fresh” to mean conducting community forums afresh, then the authors need to insert “[sic]” after the word to show that it was the participant’s mistake (i.e. we cannot use the word “fresh” as a verb). If this was an artifact of transcription, then the authors should change “fresh” to “conduct afresh”.
Thank you for highlighting this. This has been edited from the transcript to read, “*but now you have to have fresh community participation [engagement] forums (Ministry of Youth and Gender; IDI_9).* The second “have” had been dropped in transcription.

7) Discussion, fourth paragraph, fourth statement (page 27): Change “from 1990 levels by 2015” to “by 2015 from 1990 levels” for the statement to read well. This has been edited
It now reads, “to reduce maternal mortality by three quarters by 2015 from 1990 levels and a set of solutions…”

**Reviewer #2: These were my initial comments that were not forwarded to the authors:**

"I find this to be an interesting paper and the authors have written it in very clear and easy to read language. Overall, I believe the manuscript, when published will contribute to the understanding of the policy-making and agenda-setting processes. It highlights some of the controversies around adolescence, including the definition of who is an adolescent and the age to include in that definition, the acceptability of providing comprehensive sexuality education to adolescents, the problem of acceptable language, the difficulty of civil society coordination, and the perennial problem of donor-influence in agenda-setting. There are some areas I think the work may require some revisions:

1. State and non-state actor -- It will be useful in the methods section to highlight who the state actors are. I understand that this is in the Table 1, but a high level statement to show the state actors are will be useful rather than expecting the readers to wait until they get to the table or to flip to the table to find the actors and flip back to continue reading.
We have now included the statement: State actors that were targeted included government officials from the ministries of health, youth and gender affairs, devolution and planning, and education.

2. What do the notations ID1, ID2, ID3... just before a verbatim quotation mean? It lists respondents who had very similar thoughts on the topic.

First, what is the meaning of "ID"? It means In-depth-interview. We have changed it to IDI throughout the text for clarity.

Second, is the listing various IDs before a verbatim quotation indicative of the relevance of the interviews for a particular participant to the point being made? The listing indicates other respondents who agree with the point being made and had very similar views to the respondent cited. If it is, it will be necessary to state that that is the case. We have included a sentence in the first paragraph under results (page 21) that reads, “The listing of various IDIs before a verbatim quotation correspond to respondents who had similar views to the point being made.”

I think the referencing for the quotes can be improved by actually stating the respondent, for example, "Ministry of Youth" or some other form of referencing the quotes.

We have now included the type of respondents such as Ministry of Youth or International NGO.

3. In a few instances, there are cases of misplaced punctuation marks, which need to be revised. For example before a verbatim quote the authors often place a full-stop and open a bracket to place the ID1, ID2... in without a full-stop at the end of the list.

We have revised the punctuation to place the full-stop after the closed bracket throughout the paper.

4. It is surprising that the study could find that there are no adolescent SRH champions in Kenya. I wonder if this is simply a matter of the participants that were interviewed. It is really hard to believe that there are no champions. Could the author comment further on this?"

The term policy champion here is used to mean policy entrepreneur as originally defined by John Kingdon. We have changed champion to read entrepreneur throughout the text and provided some context on page 13, which references Kingdon, “Policy entrepreneurs do the process of connecting the problem with a policy solution and the political factors. The entrepreneurs 1) highlight indicators of the problem to dramatize it, 2) Push for one kind of problem definition over another – invite policymakers to see for themselves, and 3) present specific policies as the solution to a problem on the agenda 4) “Soften up” by writing papers, giving testimony, holding hearings or getting press coverage [43].

My own thinking regarding this question is in line with what Kingdon says—that entrepreneurs do more than push, push, and push for their proposals or for their conception of problems. They also lie in wait – for a window to open. In the process of leaping at their opportunity, they play a central role in coupling the streams at the window.” This kind of person has not yet emerged in the Kenyan adolescent SRH space. Typically this can be someone like the first lady, a charismatic politician with a personal history or a local or international celebrity. My thoughts are that the potential entrepreneurs e.g. politicians are too risk averse to engage with a controversial issue (page 26). It is easier to champion improved access to maternal services and providing fistula services as the first lady of Kenya has done with the beyond zero campaign but without mentioning the adolescent and “hoping/crossing fingers” that they get addressed
